# Fostering Caring Attributes to Improve Patient Care in Nursing Through Small-Group Work: Perspectives of Students and Educators

**DOI:** 10.3390/nursrep15010010

**Published:** 2025-01-03

**Authors:** Florence Mei Fung Wong

**Affiliations:** School of Nursing, Tung Wah College, Hong Kong SAR, China; florencewong@twc.edu.hk; Tel.: +852-3468-6838

**Keywords:** caring attributes, small-group work, nursing professionalism, nursing education

## Abstract

**Background**: Nursing relies on the development of caring attributes to uphold exceptional standards of care. While small-group work is a common practice in nursing education, its pivotal role in nurturing these attributes often remains underexplored. **Aim**: This study explored how caring attributes emerge in small-group settings from the perspectives of nursing students and educators. **Methods**: This qualitative study conducted semi-structured group interviews separately for students and educators. Thirteen nursing students and ten educators at a professional educational institution were interviewed. **Results**: Through the interviews, four key sets of caring attributes nurtured through small-group work were identified: interpersonal communication with respect; compassion and empathy; competence and confidence; and accountability to commitment. These findings, based on the perspectives of students and educators, underscore the essential role of caring in nursing. They emphasize how collaborative group work can serve as a catalyst for the development of these vital attributes through meaningful interpersonal interactions. Notably, the cultivation of respectful communication skills among students emerged as pivotal for enriching interactions with healthcare professionals, patients, and families, ultimately enhancing the quality of care provided. By providing a platform for interactive learning and continual practice, small-group work facilitates the internalization of these caring attributes, nurturing nursing professionalism over the course of students’ careers. **Conclusions**: This study offers invaluable insights into the profound impact of small-group work in fostering caring attributes and propelling advancements in nursing education and practice. By enhancing the development of these attributes, small-group work contributes to the delivery of compassionate and high-quality patient care.

## 1. Introduction

Caring, a fundamental aspect of humanity [1], has long been recognized as a cornerstone of nursing practice. Florence Nightingale’s pioneering work emphasized the importance of compassion and empathy in healthcare [2]. The exploration of caring within the nursing profession has revealed its intricate and multifaceted nature, encompassing a rich tapestry of attributes essential for patient well-being and recovery [3,4]. Roach [1] delineated caring attributes integral to nursing professionalism, sparking a growing recognition of their essential role in shaping patient care standards in clinical settings. These caring attributes play a crucial role in enhancing the quality of care delivered to patients, highlighting the need for their cultivation and reinforcement among nursing professionals [5,6].

In nursing, caring embodies a humanistic approach that intertwines with professional knowledge, clinical expertise, and moral values [1,7]. It establishes nurturing relationships between nurses and patients, fostering healing environments and holistic well-being [7,8]. Drawing from the insights of Watson’s seminal work, caring is not merely a task to be completed but rather a fundamental way of being that establishes nurturing relationships between nurses and patients, fostering environments that facilitate healing and holistic well-being [7,8].

In contemporary nursing education, small-group work fosters knowledge acquisition and skill development and prepares students for collaborative practice in diverse healthcare environments [9,10,11,12]. Despite its widespread adoption, there exists a gap in understanding how caring attributes are nurtured and developed through engagement in small-group activities. This study aimed to explore the evolution of caring attributes among nursing students within the context of small-group interactions. By exploring the perspectives of both undergraduate nursing students and educators, this research also illuminated the interplay between small-group dynamics and the cultivation of caring attributes. Understanding the experiences and challenges in collaborating teamwork settings can enhance pedagogical practices and develop compassionate, skilled nursing professionals for diverse healthcare settings.

The significance of caring in nursing practice transcends mere assistance and support; it extends to the core of fostering meaningful relationships that play a crucial role in enhancing patient well-being and aiding in their recovery from illness and disability. This emphasis on caring not only positively impacts healthcare services but also underscores nursing as a nurturing profession that places compassionate care at the forefront of its core values [8,9,10,11]. Without caring, there is risk in nursing practice regarding fulfilling basic accountability and understanding patients’ needs for ethical and appropriate care [12]. In an era marked by rapid technological advancements in healthcare, nurses are tasked with balancing technological proficiency with the provision of compassionate care, recognizing that the capacity for caring significantly contributes to patients’ coping mechanisms, overall well-being, and the personal and professional fulfilment of nurses themselves [3,4].

The guidance provided to nursing students as they navigate their journey towards becoming caring professionals plays a pivotal role in shaping the quality of patient care delivered in contemporary healthcare settings. The imperative to cultivate caring abilities in nursing students is crucial in evolving healthcare landscapes. A recent study demonstrated that most nursing students possessed inadequate caring skills and recommended enhancing caring education in nursing curricula [13]. Importantly, caring develops through social interaction, and collaborative teamwork settings provide the necessary environment for social interaction and collaboration to foster effective learning attributes and experiences [13,14].

Compared to lecture-based teaching, small-group teaching approaches offer a more fruitful environment for student learning in healthcare professional education [15,16,17]. Small-group work provides a student-centered model for developing essential competencies, fostering personal and professional development. Students learn from collaboration with others to enhance their knowledge, foster their social and teamwork skills, and practice their presentation about their self-learning within a group [16,17,18]. Thus, this teaching-learning approach is widely adopted in nursing education.

While previous studies have explored caring attributes development across various teaching methodologies, knowledge of the role of small-group work in nurturing caring attributes in undergraduate nursing students remains underexplored. This study aimed to illuminate this process by investigating how nursing students cultivate caring attributes through active participation in small-group activities. By shedding light on this transformative process, educators can provide tailored support to students, thereby enhancing their capacity for compassionate care and strengthening nursing professionalism within the student cohort. By examining the impact of small-group interactions on the development of caring attributes, educators can better understand how to foster a caring ethos among future nursing professionals.

## 2. Materials and Methods

### 2.1. Design

A qualitative research design was employed, utilizing semi-structured group interviews as the primary data collection method. The Consolidated Criteria for Reporting Qualitative Research (COREQ-32) [19] (see Supplementary Materials S1 ) guided the study process to ensure that essential components are reported with sufficient information to improve the quality and transparency of a qualitative research. The COREQ-32 consists of three domains, including research team and reflexivity (Domain 1), study design (Domain 2), and data analysis and findings (Domain 3). In Domain 1, the sub-domains are personal characteristics (five items) and relationship with participants (three items). In Domain 2, the sub-domains are theoretical framework (one item), participant selection (four items), setting (three items), and data collection (seven items). In Domain 3, the sub-domains are data analysis (five items) and reporting (four items).

### 2.2. Sample

The study participants included undergraduate nursing students from various cohorts and nurse educators from a professional educational institution who had experience in learning or teaching within small-group settings. The selection of participants was purposive in nature to ensure relevance to the study objectives.

### 2.3. Data Collection

Semi-structured face-to-face group interviews were conducted by the principal investigator (PI) in a conducive environment within the study institute. Prior to the interviews, participants were briefed on the study objectives and provided with informed consent forms. Students and educators were interviewed separately in sessions consisting of three to four individuals to encourage open and uninhibited expression of viewpoints. Open-ended questions were posed to elicit participants’ perspectives on the caring attributes essential for your nursing profession. The students were given the following prompt: “Share how you develop your caring attributes through working in a small group for your nursing profession”. The educators were given the following prompt: “Share how students can develop caring attributes through working in a small group for their nursing profession”. These prompts facilitated the focus group interviews according to the provided interview guide to support participants’ willingness to share their authentic experiences in a peer-influenced and encouraging environment. This approach also minimized biases and ensured that valuable and pertinent perspectives were gathered from participants to address the study objectives. The interview guide for both students and educators can be found in Supplementary Materials S2.

Each interview had a duration of around 45 to 60 min and was recorded digitally. Data saturation was reached, and member checks were conducted to verify the accuracy of their experiences at the conclusion of each interview.

### 2.4. Ethical Considerations

Ethics approval (2015-00-55 R150401) was obtained from the research ethics committee of the educational institute before commencing the study. Participants were required to sign written consent forms prior to their involvement in the study, ensuring confidentiality and anonymity of all information shared.

### 2.5. Data Analysis

After each interview, the research assistant (RA) transcribed the audio-recorded interviews verbatim, and the principal investigator (PI) initially reviewed the content to confirm accuracy. Data analysis was conducted using the Colaizzi method [20]. Both the PI and RA independently reviewed and analyzed the transcripts to pinpoint significant statements related to caring attributes. A meticulous line-by-line approach was employed to create codes, which were then categorized thematically. Triangulation was utilized to cross-reference and validate data, enhancing the reliability of the findings. To mitigate biases and ensure fidelity to the participants’ perspectives, bracketing and member checking techniques were applied.

## 3. Results

In this study, 13 nursing students were in year 2 to year 5 of their bachelor’s degree in nursing, and they had previous experience in collaborating on group projects to address specific health issues across different courses. These group projects encompassed a variety of formats, including written assignments as well as presentations in formats such as PowerPoint, posters, videos, and community-based projects. The 10 nurse educators had from 2 to more than 30 years’ teaching experience. The participants were divided into four groups of students and three groups of educators, with each group consisting of three to four participants. The study identified caring attributes based on the perspectives of the participants, including interpersonal communication with respect, compassion and empathy, competence and confidence, and accountability to commitment.

### 3.1. Interpersonal Communication with Respect

Interpersonal communication plays a vital role in fostering healthy group dynamics [21,22]. Respect is highlighted as a crucial element in creating a positive work culture, as it enhances mutual trust, understanding, and productivity [23]. Effective interpersonal communication with respect was found to enable students to form closer relationships and cultivate a peaceful learning environment [23,24,25]. All participants emphasized the significance of respectful interpersonal communication in facilitating idea sharing and relationship-building within the group. They acknowledged that communication skills are essential for clinical practice and require continuous learning and practice.

Student #4 shared, “*Working in a group facilitates our communication among our teammates. I can communicate with my teammates to share our viewpoints and to share our feelings. I found that my communication skills have improved each time through sharing and listening to others with respect and understanding*”.

Student #11 highlighted, “*We not only learn knowledge and practical skills but also how to communicate with others. In our study, we need to do group works in many courses. Through working with others, I understand how to communicate with others in an appropriate way…. It is the practice of communication with respect. If no respect, the communication is failure*”.

Student #6 expressed, “*Small-group work allows me to know new friends and communicate with them. Learning in a group helps me talk with patients and their families in clinical practice*”.

All educators emphasized the importance of developing students’ communication skills through small-group work, as effective communication is essential for providing quality patient care. They stressed the need for students to learn how to communicate effectively with team members from diverse backgrounds, interests, and personalities.

Educator #6 stated, “*Students must understand and accept others from different backgrounds and personalities. Good relationships and communication are key to success in their work. Respect is crucial for establishing and maintaining effective communication and interpersonal relationships. Some students are able to communicate with others very well. They listen to their groupmates, and they share their viewpoints. I believe that communication with patience, acceptance, and respect is very important for an effective communication*”.

Educator #1 noted, “*Most students learn communication skills while working with their teammates. Communication is vital for their future clinical practice. Mutual respect and understanding are essential components of effective communication. Communication gaps or misunderstandings can hinder group work, highlighting the importance of mutual respect in fostering effective communication*”.

### 3.2. Compassion and Empathy

Compassion is a fundamental humanistic trait that involves showing consideration for others through empathy, sympathy, respect, and dignity [26,27]. A compassionate individual actively engages in understanding and alleviating another person’s suffering, demonstrating a willingness to learn about their experiences in order to offer assistance and support consistently [28,29,30]. Empathy, on the other hand, is the ability to recognize and comprehend an individual’s distress, playing a vital role in fostering therapeutic relationships [26,31]. An empathetic connection involves a genuine desire to comprehend a person’s needs and emotions [32]. Both compassion and empathy are essential elements for building trust and establishing meaningful connections with others [26,28]. Many students emphasized the importance of understanding their groupmates’ needs, abilities, and interests before delegating tasks. They preferred forming groups with friends, as they were familiar with each other, making it easier to identify and address each other’s needs.

Student #4 expressed, “*We need to work together. First, to understand the needs of your teammates. Then, we can distribute our work more easily. …To me, I prefer forming a group…better with friends; we know one another. It is easier for me to ask their needs and their feelings… If anyone needs help in the team, we all should help. It is good to show our compassion by understanding the needs of others and provide timely assistance while working together*”.

Nurse educators acknowledged that students learned to demonstrate compassion and empathy by recognizing and addressing the needs of others through group work, enabling them to offer support and assistance effectively. They highlighted that small-group work served as a training ground for real-life clinical scenarios, allowing students to practice collaborating with individuals from diverse backgrounds. The teaching-learning approach in the classroom has the potential to enhance patient care and foster interdisciplinary collaboration in clinical practice. To support student learning, educators often encourage students to form groups with classmates they are already familiar with. Educators anticipate that students will grasp their teammates’ needs effectively and engage in collaborative relationships from the outset while working in a group.

Educator #8 stated, “*The purpose of small-group work goes beyond project completion. Students are encouraged to collaborate, share talents, and support each other. Interaction in small groups helps students understand the needs of others and show compassion and empathy to those in need*”.

Educator #2 added, “*Students are trained to be caring and compassionate nurses. Small-group work helps them develop these attributes by fostering a culture of care and willingness to help others*”.

### 3.3. Competence and Confidence

Competence is defined as the ability to perform tasks and fulfil duties at an excellent standard, requiring continuous study and practice [33,34,35]. Confidence, on the other hand, is closely linked to competence and is developed based on an individual’s proficiency with relevant knowledge and skills [36,37]. While competence showcases one’s potential and capabilities, confidence serves as the driving force behind competence [32].

Students reported that they benefited from self-study and group discussions during collaborative work, enabling them to enhance their competence and confidence. Both students and educators acknowledged that small-group work played a significant role in expanding knowledge and fostering collaborative relationships essential in the nursing field.

Student #12 shared, “*I love group work, as it enhances my learning efficiency. Working with friends and managing tasks within my control boosts my confidence in myself and others, leading to stronger relationships. Increased learning results in enhanced competence, which in turn builds my confidence to share knowledge with others*”.

Student #4 expressed*,* “*Group work allows for efficient learning as tasks are divided among team members, and collaboration leads to a comprehensive outcome. Learning from peers enhances my competence, crucial for patient care in clinical practice. Competence breeds confidence, especially in delivering patient care*”.

Educator #3 noted*,* “*Small-group work facilitates student learning through peer interaction, enhancing their competence and confidence. Competence is vital for students to enrich their value and confidence, preparing them for clinical practice*”.

Educator #5 emphasized, “*Learning is a process. Competence is key to becoming qualified nurses who provide exceptional patient care. Caring, a fundamental aspect of nursing, is enriched by competence, which boosts confidence and the ability to care for those in need*”.

### 3.4. Accountability to Commitment

Accountability is the sense of fulfilment in a task, indicating an individual’s willingness to bear responsibility and take action [38,39]. Commitment, on the other hand, involves devoting oneself to executing best practices and delivering care of superior quality [40,41,42]. Accountability and commitment are interconnected, with accountable individuals taking responsibility for tasks and committing to achieving productive outcomes [38,43]. During group work, students experience a heightened sense of shared accountability, leading to increased individual commitment and improved group interactions [44,45]. Students unanimously agreed that they were responsible for their assigned tasks and recognized the importance of personal commitment to learning for better outcomes.

Student #5 expressed, “*Learning is a personal asset. Putting effort and commitment into learning is essential for personal growth. Each group member should focus on their tasks to contribute to a successful outcome. The more responsibility I have, the more dedicated I become*”.

Student #7 shared, “*In group projects, we distribute tasks among team members. Each of us takes ownership of our assigned parts, ensuring that we contribute effectively to the project. Responsibility involves self-investment, and the team benefits when every member fulfils their role diligently*”.

The educators observed that students needed to actively engage in their learning and collaborate with their peers in group settings. Students were accountable for their individual and group learning processes to achieve desired outcomes, fostering a sense of commitment.

Educator #11 emphasized, “*Small-group work is designed to teach students the importance of collaboration, responsibility, and communication. Students must respect and cooperate with one another to enhance their personal and professional growth. Taking accountability and showing commitment in group work is crucial for students to proactively engage in learning and take ownership of their educational journey*”.

In summary, students effectively cultivate four core caring attributes through small-group work: interpersonal communication marked by respect; compassion and empathy; competence and confidence; and accountability to commitments. These caring attributes are essential for enhancing professional values, promoting interdisciplinary collaboration, and ultimately improving patient outcomes in the field of nursing.

## 4. Discussion

**Figure 1:**
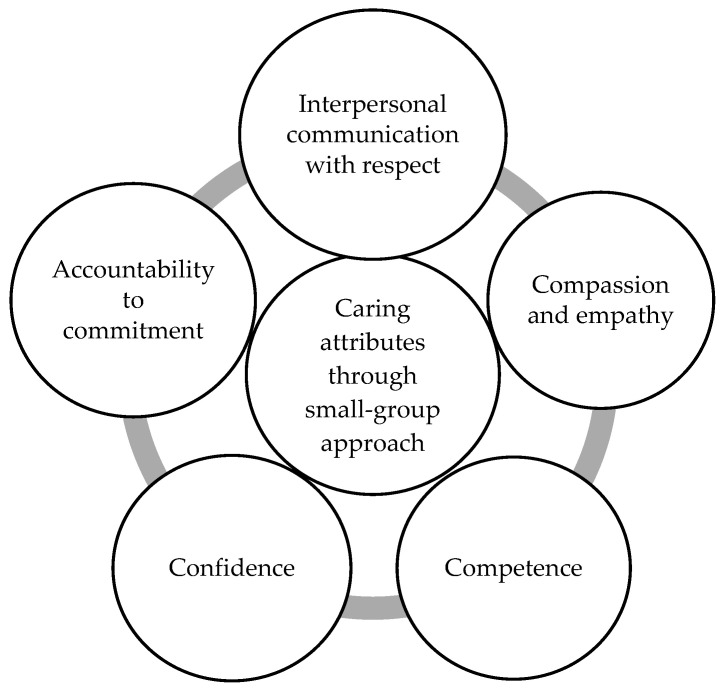
Illustrates the interplay among these caring attributes nurtured through collaborative group work.

Nursing encompasses essential caring attributes that are fundamental to nursing professionalism. These attributes are crucial elements for providing cost-effective and appropriate patient care [3]. Caring is viewed as an interpersonal process that integrates professional knowledge, skills, and sensitivity to address the bio-psycho-social needs of individuals and deliver suitable treatment and care [46]. Small-group activities offer a platform for students to practice and refine these essential qualities in a supportive and collaborative environment. Through engaging in discussions, problem-solving tasks, and reflective exercises within small groups, nursing students can enhance their communication skills, emotional intelligence, and capacity for empathetic care [14,18]. The study emphasizes the significance of collaborative work in enabling students to identify learning needs, establish interpersonal relationships, share experiences, collaborate, and provide support, ultimately fostering the development of caring attributes. The four caring attributes identified through small groups include interpersonal communication with respect, compassion and empathy, competence and confidence, and accountability to commitment. These caring attributes are interconnected, with interpersonal communication grounded in respect serving as the foundational attribute linking the others. Figure 1 illustrates the interplay among these caring attributes nurtured through collaborative group work. Small-group work serves as an effective educational platform for nursing students to not only meet their learning objectives but also cultivate their caring attributes, enhancing the quality of care and fostering the development of nursing professionalism.

**Interpersonal communication with respect**

Based on the perspectives of students and educators, four sets of interconnected caring attributes were identified. Interpersonal communication with respect emerges as a crucial factor that links and activates other caring attributes. Watson’s caring model underscores the importance of forming connections with others to cultivate caring moments and develop relationships [47]. Respectful communication is essential for nurturing students’ collaborative skills, building trust, and fostering authentic caring relationships [47,48]. Effective communication within groups enhances productivity, quality outcomes, and conflict resolution, thereby contributing to a harmonious learning environment and the establishment of therapeutic relationships [22,23,24].

Through effective interpersonal communication, which involves exchanging learning experiences, providing feedback, and engaging in discussions within a group setting, students can enhance both individualized and group learning experiences [21,22,23,24,25]. Watson’s caring model underscores the importance of establishing connections with others to create caring moments and cultivate transpersonal relationships [47]. Effective interpersonal communication with respect was emphasized as crucial for fostering healthy group dynamics and creating a positive work culture [22,23,24]. This, in turn, paves the way for the development of other essential caring attributes [49,50,51]. Participants noted that respectful communication enables closer relationships, idea sharing, and a peaceful learning environment. Developing communication skills through small-group work was seen as essential for clinical practice success. Collaborative teamwork, aimed at reaching mutually beneficial agreements, can be honed through effective interpersonal communication, transcending individual differences in personalities, capabilities, and cultural backgrounds [23,24].

**Compassion and empathy**

Compassion plays a pivotal role in enhancing team collaboration and fostering harmonious work environments [26,27,28]. In successful teams, communication thrives with close interactions rooted in compassionate and empathetic care among team members [52,53]. Students emphasized the importance of mutual consideration and understanding in team collaborations, highlighting the significance of forming relationships based on these values [18,23]. The study revealed that students, through group work, exhibit a heightened awareness of their peers’ needs, indicating the development of compassion and empathy within the team. Compassion and empathy, serving as catalysts for enhancing interpersonal communication and fostering collaborative relationships within the team were identified as fundamental humanistic traits necessary for building trust and meaningful connections and providing compassionate care [50,51,52]. Students preferred forming groups with friends to better understand each other’s needs and offer support. Educators highlighted that small-group work serves as a training ground for demonstrating compassion and empathy in real-life clinical scenarios.

**Competence and confidence**

Competence encompasses the acquisition and application of evidence-based knowledge and skills in therapeutic interventions for current patient care [46,47]. Given the high expectations in healthcare services, nursing students must possess adequate competence to ensure safe patient care [18,22,46]. Nurses are expected to utilize updated knowledge and skills to deliver competent and quality healthcare services [34]. In the study, students enhanced competence through interactions with others, engaging actively in self-study, reflections, group discussions, feedback, sharing, and clarifications to facilitate effective learning and improvement opportunities [35,36]. Students engage in self-directed learning and group learning from sharing and discussions to seek more learning opportunities for improvement and excellence [16,17,18]. It is apparent that competence is reinforced over time through the engagement of lifelong learning and day-to-day practice [44,45]. In that sense, competence is essential in students’ lifelong learning for ensuring competent practice in the long term. Importantly, competence and confidence are interrelated and exist simultaneously [34]. Where competence reflects an individual’s potential and capabilities, confidence acts as the motivation to drive competence [54,55]. Confidence is developed in relation to the individual’s ability and competence with relevant knowledge and skills [1]. A confident person will make changes using knowledge and skills for better outcomes and address difficulties with a sense of commitment. Based on the participants’ experience, they admitted competence enhancement following an increase in confidence in performance and practice, ultimately expanding knowledge and fostering collaborative relationships essential in the nursing field.

**Accountability to commitment**

Accountability and commitment were emphasized as interconnected traits that lead to improved group interactions and outcomes. In the present study, accountability is a key focus and is cultivated among students while working in groups. Accountability entails individual responsibility and commitment and reflects conscience [54,55,56]. Conscience is the innate sense of right and wrong, guiding individuals’ moral, ethical, and legal values in their actions and decisions [57]. It motivates individuals to exert effort and engage in responsible tasks, making moral judgments based on their cultural and religious background, education, life experiences, and knowledge and skills in relevant situations [56]. Through interactions with peers, students develop a heightened conscience and a stronger sense of moral responsibility [42,43]. They actively participate in group tasks, identifying and refining their values and fostering moral accountability [43,57]. In this context, conscience serves as a motivating factor for individual accountability, where accountability implies personal commitment.

Commitment involves a willingness to make sacrifices and dedicate oneself to achieving optimal practices and progress [44,45]. It is tied to an individual’s attitude and efforts to deliver high-quality or exceptional care [49,56]. Therefore, commitment encompasses a significant level of individual responsibility, willingness, and loyalty to strive for continuous improvement and the delivery of therapeutic care [49,57,58]. By engaging with peers, students can cultivate a sense of commitment through self-directed learning and active participation in group discussions and activities. Notably, students recognized the importance of personal commitment to learning and taking responsibility for their assigned tasks during group work. Students demonstrated a willingness to commit to collaborative efforts with their peers, influenced by their relationships within the group, personal interests, capabilities, and the complexity of the tasks at hand [16]. Educators stressed the significance of collaboration, responsibility, and communication in fostering student growth and engagement in the learning process [22,59]. Therefore, fostering collaborative team dynamics involves mutual respect, harmonious teamwork, individual and collective commitment, and collaborative partnerships.

In essence, cultivating caring attributes is an ongoing journey of personal and professional development aimed at enhancing the fulfilment of both learning and practical experiences [56,59,60]. The study findings demonstrated that small-group work in nursing education effectively cultivates caring attributes such as interpersonal communication with respect; compassion, and empathy; competence and confidence; and accountability to commitments. These attributes are essential for enhancing professional values, promoting interdisciplinary collaboration, and ultimately improving patient outcomes in the field of nursing. Therefore, this study’s results contribute to the existing body of literature by providing specific insights and perspectives from nursing students and educators on how small-group work can enhance caring attributes such as interpersonal communication, compassion, empathy, competence, confidence, and accountability. While the concepts of communication, compassion, competence, confidence, and accountability have been studied in various contexts before, the unique contribution of this study lies in its focus on how these attributes are developed and nurtured through small-group work, specifically within the nursing education setting. The study sheds light on the experiences, perspectives, and practices of nursing students and educators, highlighting the importance of collaborative learning environments in fostering these essential caring elements. This study also provides valuable insights into the role of small-group work in promoting these attributes among nursing students and educators, offering a nuanced understanding of their development in the context of nursing education.

### 4.1. Limitations

The present study utilized a qualitative design with in-depth interviews to investigate the development of caring attributes through small-group work from the viewpoints of nursing students and educators. However, the findings are based on the subjective experiences of the participants, limiting their generalizability to other professional groups or settings. Additionally, institutional rules and regulations may have influenced these experiences. Given the diverse backgrounds and experiences of participants in small-group work, including study duration, group dynamics, and learning expectations, perspectives may vary significantly. Therefore, conducting large-sample quantitative studies can provide further insights into the cultivation of caring attributes through different educational approaches, such as small-group work.

### 4.2. Implications for Practice

Interpersonal communication with respect is fundamental in fostering a collaborative work environment and promoting professional caring practices, aligning with the emphasis on multidisciplinary teamwork in healthcare settings [23,48]. Effective and respectful communication not only enhances patient–nurse and interdisciplinary relationships but also drives the development of other essential caring attributes, such as compassion, empathy, competence, confidence, accountability, and commitment, ultimately ensuring safe and optimal patient care [40,61]. Therefore, nurturing caring attributes is critical for shaping nursing values and enhancing patient outcomes in clinical practice.

The study emphasizes the importance of integrating interactive learning approaches, such as small-group work, into the nursing curriculum to facilitate the development of caring qualities. To facilitate students’ development of caring attributes, nurse educators should integrate interactive learning approaches, such as small-group work, into the curriculum. Interactive learning methods not only enhance students’ knowledge and practical skills but also foster personal and professional attributes through collaborative learning experiences, which are particularly beneficial for nursing practice [18,22]. Nurse educators should closely monitor students’ progress in developing caring qualities, offering guidance and support during group work [18,60]. Given the positive impact of interactive learning on the development of caring attributes and nursing professionalism, integrating small-group work and other interactive approaches into theoretical and clinical nursing courses is recommended. Nurse educators play a crucial role in monitoring students’ progress in developing caring attributes and offering guidance and support during group work. Educators are responsible to ensure that students are well equipped to provide safe and optimal patient care in clinical practice.

The present study raises awareness among nurse educators about the significance of fostering caring attributes through interactive teaching–learning methods. Program developers are encouraged to consider incorporating diverse interactive teaching–learning strategies, including small-group work, into nursing curricula to enhance caring attributes and promote the development of nursing professionalism. This highlights the need for ongoing curriculum development that prioritizes the cultivation of essential caring attributes in nursing students. Emphasizing caring attributes in nursing practice is essential for ensuring patient safety, facilitating patient recovery, and upholding the standards of healthcare services. By focusing on the development of caring attributes through interactions in classroom settings, educators and students can collectively work towards enhancing nursing professionalism and optimizing patient care outcomes.

The strengths of this qualitative study lie in its ability to offer valuable insights into the development of caring attributes through small-group approaches. Through exploring the perspectives of both students and educators, the study sheds light on how caring skills can be effectively nurtured through collaborative group work. However, it is important to note that the generalizability of the study findings may be limited, as all participants, both students and educators, were recruited from the same educational institution, impacting the transferability of the results to other institutions with different organizational operations and curriculum structures. Moreover, all participating students and educators expressed positive views regarding the effectiveness of small-group approaches, which greatly influenced the predominantly positive outcomes emphasized in this research. To further enhance understanding of the development of caring attributes, it is advisable to conduct a mixed-method study with a diverse range of participants. This approach would provide a comprehensive exploration of caring attributes and allow for the evaluation of these attributes using quantitative methods.

## 5. Conclusions

The essence of caring in nursing practice transcends mere tasks and procedures, embodying the very humanity of nurses and extending to embrace the humanity of those under their care. It serves as the cornerstone of fostering mutual and trusting human-to-human relationships between nurses and patients, ultimately enhancing the quality of patient care in nursing. The cultivation of caring attributes among nursing professionals is paramount in embodying core professional values and elevating nursing professionalism to new heights. The emphasis on caring in nursing practice is indispensable for ensuring patient safety, promoting patient recovery, and upholding the standards of healthcare services. This study underscores the pivotal role of students in developing caring attributes through meaningful interactions in classroom settings, highlighting the significance of adequate teaching resources, such as involving qualified teaching staff in small-group approaches, to facilitate effective group dynamics and interactions. Both educators and students recognize and appreciate the benefits of small-group work in nurturing and enhancing of caring attributes. The identified caring attributes, which include interpersonal communication with respect, compassion, empathy, competence, confidence, accountability, and commitment, are essential in nursing professional development and in optimizing patient care.

## Data Availability

The data presented in this study are available on request from the corresponding author. The data are not publicly available to maintain confidentiality.

## Supplementary Materials

The following supporting information can be downloaded at https://www.mdpi.com/article/10.3390/nursrep15010010/s1, S1: The Consolidated Criteria for Reporting Qualitative Research (COREQ-32); S2: Interview guide.

## Public Involvement Statement

There was no public involvement in any aspect of this research.

## Guidelines and Standards Statement

A complete list of reporting guidelines can be accessed via the equator network: https://www.equator-network.org/.

## References

[1] Roach M.S. (1992). The Human Act of Caring: A Blueprint for the Health Professions, Revised Ed..

[2] Nightingale F. (1992). Notes on Nursing.

[3] Nadelson S.G., Zigmond T., Nadelson L., Scadden M., Collins C. (2016). Fostering caring in undergraduate nursing students: An integrative review. J. Nurs. Educ. Pract..

[4] Wysong P.R., Driver E. (2009). Patients’ perceptions of nurses’ skill. Crit. Care Nurse.

[5] Blasdell N. (2017). The Meaning of Caring In Nursing Practice. Jnt. J. Nurs. Clin. Pract..

[6] Calong K.A.C., Soriano G.P. (2018). Caring Behavior and Patient Satisfaction: Merging for Satisfaction. Int. J. Caring Sci..

[7] Watson J. (2002). Assessing and Measuring Caring in Nursing and Health Sciences.

[8] Watson J. (1988). Nursing: Human Science and Human Care. A Theory of Nursing.

[9] Ozgonul L., Alimoglu M.K. (2019). Comparison of lecture and team-based learning in medical ethics education. Nurs. Ethics.

[10] Drahošová L., Jarošová D. (2016). Concept caring in nursing. Cent. Eur. J. Nurs. Midwifery.

[11] Wilkin K. (2003). The meaning of caring in the practice of intensive care nursing. Br. J. Nurs..

[12] Karlsson M., Pennbrant S. (2020). Ideas of caring in nursing practice. Nurs. Philos..

[13] Bagnall L.A., Taliaferro D., Underdahl L. (2018). Nursing students, caring attributes, and opportunities for educators. Int. J. Hum. Caring.

[14] Burgess A., van Diggele C., Roberts C., Mellis C. (2020). Facilitating small group learning in the health professions. BMC Med Educ..

[15] Mennenga H., Smyer T. (2010). A model for easily incorporating team based learning into nursing education. Int. J. Nurs. Educ. Scholarsh..

[16] Wong F.M.F., Kan C.W.Y. (2022). Online Problem-Based Learning Intervention on Self-Directed Learning and Problem-Solving through Group Work: A Waitlist Controlled Trial. Int. J. Environ. Res. Public Health.

[17] Wong M.F.F. (2020). Development of Higher-Level Intellectual Skills through Interactive Group Work: Perspectives between Students and Educators. Med. Clin. Res..

[18] Wong F.M.F. (2018). A phenomenological research study: Perspectives of student learning through small group work between undergraduate nursing students and educators. Nurse Educ. Today.

[19] Tong A., Sainsbury P., Craig J. (2007). Consolidated criteria for reporting qualitative research (COREQ): A 32-item checklist for interviews and focus groups. Int. J. Qual. Health Care.

[20] Colaizzi P.F., Valle R. (1978). Psychological research as the phenomenologist views it. Existential Phenomenological Alternatives for Psychology.

[21] Vertino K. (2014). Effective Interpersonal Communication: A practical guide to improve your life. Online J. Issues Nurs..

[22] Wong M.F.F. (2017). A cross-sectional study: Collaborative learning approach enhances learning attitudes of undergraduate nursing students. GSTF JNHC.

[23] Krep G.L. (2016). Communication and effective interprofessional health care teams. Int. Arch. Nurs. Health Care.

[24] Kourkouta L., Papathanasiou I.V. (2014). Communication in nursing practice. Mater Socio-Medica.

[25] Gregory J. (2024). Understanding the communication skills that support nurses to provide person-centred care. Nurs. Stand..

[26] Bramley L., Matiti M. (2014). How does it really feel to be in my shoe? Patients’ experiences of compassion within nursing care and their perceptions of developing compassionate nurses. J. Clin. Nurs..

[27] Babaei S., Taleghani F., Farzi S. (2022). Components of Compassionate Care in Nurses Working in the Cardiac Wards: A Descriptive Qualitative Study. J. Caring Sci..

[28] Brennan M. (2006). An evaluation of perceived education and training needs of staff nurses and care officers. J. Forensic Nurs..

[29] von Dietze E.V., Orb A. (2000). Compassionate care: A moral dimension in nursing. Nurs. Inq..

[30] Watts E., Patel H., Kostov A., Kim J., Elkbuli A. (2023). The Role of Compassionate Care in Medicine: Toward Improving Patients’ Quality of Care and Satisfaction. J. Surg. Res..

[31] Reynolds W. (2000). The Measurement and Development of Empathy in Nursing.

[32] Clark C.M. (2018). In Pursuit of empathy. Reflections on Nursing Leadership.

[33] Canadian Holistic Nurses Association (2024). Caring: The Core of Nursing Practice. https://www.chna.ca/caring-the-core-of-nursing-practice-via-hurst-review/.

[34] Clanton J., Gardner A., Cheung M., Mellert L. (2014). The relationship between confidence and competence in the development of surgical skills. J. Surg. Educ..

[35] Tabari-Khomeiran R., Kiger A., Para-Yekta Z., Ahmadi E. (2007). Competence development among nurses: The process of constant interaction. J. Contin. Educ. Nurs..

[36] Rautava V.P., Palomäki E., Innamaa T., Perttu M., Lehto P., Palomäki A. (2013). Improvement in self-reported confidence in nurses’ professional skills in the emergency department. Scand. J. Trauma Resusc. Emerg. Med..

[37] Suandika M., Tang W.R., Ulfah M., Cahyaningrum E.D. (2021). self-confidence of nurses philosophy: A concept analysis. Open Access Maced. J. Med. Sci..

[38] Bergman R. (1981). Accountability–Definition and dimension. Int. Nurs. Rev..

[39] Zaitoun R.A., Said N.B., de Tantillo L. (2023). Clinical nurse competence and its effect on patient safety culture: A systematic review. BMC Nurs..

[40] Al-Hamdan Z., Dalky H., Al-Ramadneh J. (2017). Nurses’ Professional Commitment and Its Effect on Patient Safety. Glob. J. Health Sci..

[41] Aminuddin A., Musrah S., Wijayanti L.A., Utama Y.A., Suprapto (2023). Commitment and Job Satisfaction with Nurse Job Performance. J. Nurs. Pract..

[42] Baillie L. (2017). An exploration of the 6Cs as a set of values for nursing practice. Br. J. Community Nurs..

[43] Dohmann E. (2009). Accountability in Nursing. Six Strategies to Build and Maintain a Culture of Commitment.

[44] Lawler E.J., Thye S., Yoon J. (2008). Commitment in structurally enabled and induced Exchange Relations. Soc. Psychol. Q..

[45] Kaldal M.H., Voldbjerg S.L., Grønkjaer M., Conroy T., Feo R. (2024). Newly graduated nurses’ commitment to the nursing profession and their workplace during their first year of employment: A focused ethnography. J. Adv. Nurs..

[46] Finfgeld-Connett D. (2008). Meta-synthesis of caring in nursing. J. Clin. Nurs..

[47] Watson J., Parker M.E. (2001). Jean Watson: Theory of human caring. Nursing Theories and Nursing Practice.

[48] Lee A., Doran D. (2017). The role of interpersonal relations in healthcare team communication and patient safety: A proposed model of interpersonal process in teamwork. Can. J. Nurs. Res..

[49] Nesje K. (2016). Personality and professional commitment of students in nursing, social work, and teaching: A comparative survey. Int. J. Nurs. Stud..

[50] Sprecher S., Fehr B. (2005). Compassionate love for close others and humanity. J. Soc. Pers. Relat..

[51] Kondaguli S.V., Rana S., Balagar N. (2023). Compassionate nursing care: A refined approach to facilitate healing—An expository perspective review. Int. J. Health Sci. Res..

[52] Betcher D.K. (2010). Elephant in the room project: Improving caring efficacy through effective and compassionate communication with palliative care patients. Medsurg Nurs..

[53] Sharkiya S.H. (2023). Quality communication can improve patient-centred health outcomes among older patients: A rapid review. BMC Health Serv. Res..

[54] Serafin L., Strząska-Kliś Z., Kolbe G., Brzozowska P., Szwed I., Ostrowska A., Czarkowska-Pączek B. (2022). The relationship between perceived competence and self-esteem among novice nurses—A cross-sectional study. Ann. Med..

[55] Dehmer J.J., Amos K.D., Farell T.M., Meyer A.A., Newton W.P., Meyers M.O. (2013). Competence and confidence with basic procedural skills: The experience and opinions of fourth-year medical students at a single institution. Acad. Med..

[56] Gogola D.B. (2018). Selfless caring of theory commitment. Int. J. Nurs. Sci..

[57] Jensen A., Lidell E. (2009). The influence of conscience in nursing. Nurs. Ethics.

[58] Kalantari S., Modanloo M., Ebadi A., Khoddam H. (2024). Concept analysis of conscience-based nursing care: A hybrid approach of Schwartz-Barcott and Kim’s hybrid model. BMC Med. Ethics.

[59] Asfour H.I., Ahmed F.R., Abd El Halim G.E. (2016). Measuring changes in attitudes, practice, and knowledge of undergraduate nursing students after receiving in educational intervention in ethical comportment in critical care nursing. J. Nurs. Educ..

[60] Porr C., Egan R. (2013). How does the nurse educator measure caring?. Int. J. Nurs. Educ. Scholarsh..

[61] Wong F.M.F. (2024). Job satisfaction in nursing: A qualitative inquiry into novice and experienced nurses’ perspectives. Nurse Educ. Pract..

