# Supplementary material for: Fostering Caring Attributes to Improve Patient Care in Nursing Through Small-Group Work: Perspectives of Students and Educators
Source: Nurs Rep. 2025 Jan 3;15(1):10. doi: 10.3390/nursrep15010010 (PMC11767813; doi:10.3390/nursrep15010010)
Supplement: Supplementary file 1 [file nursrep-15-00010-s001.zip › nursrep-3306917-supplementary.pdf]

S1. Consolidated criteria for reporting qualitative studies (COREQ): 32-item checklist

No	Item	Guide questions/description	Responses
Domain 1: Research team and reflexivity			
Personal Characteristics			
1.	Interviewer/facilitator	Which author/s conducted the interview or focus group?	The PI & the research assistant
2.	Credentials	What were the researcher's credentials? E.g. PhD, MD	PI obtained Doctoral Degree
3.	Occupation	What was their occupation at the time of the study?	PI is an Associate Professor.
4.	Gender	Was the researcher male or female?	Female
5.	Experience and training	What experience or training did the researcher have?	PI had training in research methods and experience of conducting a phenomenological study with focus group interviews.
Relationship with participants			
6.	Relationship established	Was a relationship established prior to study commencement?	Educators & students or colleagues
7.	Participant knowledge of the interviewer	What did the participants know about the researcher? e.g. personal goals, reasons for doing the research	The participants were explained about the study purposes and their involvement before the commencement of the study
8.	Interviewer characteristics	What characteristics were reported about the interviewer/facilitator?	The interviewer is a nurse educator

		e.g. Bias, assumptions, reasons and interests in the research topic	and involved in teaching in small group work. She concerns about caring attributes of nursing students in their professional development.
No	Item	Guide questions/description	Responses
Domain 2: study design			
Theoretical framework			
9.	Methodological orientation and Theory	What methodological orientation was stated to underpin the study? e.g. grounded theory, discourse analysis, ethnography, phenomenology, content analysis	Yes. It is a phenomenological study. The Colaizzi's method was used to systematically analyze the transcripts.
Participant selection			
10.	Sampling	How were participants selected? e.g. purposive, convenience, consecutive, snowball	13 students and 10 educators
11.	Method of approach	How were participants approached? e.g. face-to-face, telephone, mail, email	Face-to-face focus group interviews
12.	Sample size	How many participants were in the study?	23 participants
13.	Non-participation	How many people refused to participate or dropped out? Reasons?	None
Setting			
14.	Setting of data collection	Where was the data collected? e.g. home, clinic, workplace	In the study site

No	Item	Guide questions/description	Responses
15.	Presence of nonparticipants	Was anyone else present besides the participants and researchers?	No
16.	Description of sample	What are the important characteristics of the sample? e.g. demographic data, date	Yes, the sample characteristics were reported.
No	Item	Guide questions/description	Responses
Data collection			
17.	Interview guide	Were questions, prompts, guides provided by the authors? Was it pilot tested?	Yes, open-end guided questions were provided by the PI and a rehearsal session was done before interview.
18.	Repeat interviews	Were repeat interviews carried out? If yes, how many?	No. Each group was interviewed once
19.	Audio/visual recording	Did the research use audio or visual recording to collect the data?	Audio-recording was used during interviews
20.	Field notes	Were field notes made during and/or after the interview or focus group?	The field notes were taken by the research assistant during focus group interviews.
21.	Duration	What was the duration of the interviews or focus group?	The duration of each focus interview was 45 to 90 minutes.
22.	Data saturation	Was data saturation discussed?	Yes. The interview was stopped when data saturation had been reached.
23.	Transcripts returned	Were transcripts returned to participants for comment and/or correction?	No but the PI performed the member check during the interviews. The transcripts were analyzed by the PI and the research assistant independently.

			Discussion was done until consensus was reached if discrepancies occurred.
Domain 3: analysis and findings			
Data analysis			
24.	Number of data coders	How many data coders coded the data?	Two, PI and the research assistant
25.	Description of the coding tree	Did authors provide a description of the coding tree?	No.
26.	Derivation of themes	Were themes identified in advance or derived from the data?	Themes were derived from the data.
No	Item	Guide questions/description	Responses
27.	Software	What software, if applicable, was used to manage the data?	No
28.	Participant checking	Did participants provide feedback on the findings?	The reviewer would make conclusion to confirm and validate with the participants if<br>the meaning of statements from the participants were accurate.
Reporting			
29.	Quotations presented	Were participant quotations presented to illustrate the themes / findings? Was each quotation identified? e.g. participant number	Yes
30.	Data and findings consistent	Was there consistency between the data presented and the findings?	Yes
31.	Clarity of major themes	Were major themes clearly presented in the findings?	Yes

32.	Clarity of minor themes	Is there a description of diverse cases or discussion of minor themes?	No

Interview guide

A qualitative study: comparing the perspectives on development of caring attributes in small group learning between educators and students in nursing education

Question Guide for students

Starting with the open-ended question:

Share how you develop your caring attributes through working in a small group for your nursing profession.

Probing questions:

1. With your experience of participating in a small group, what types of caring attributes can be developed through working in a small group?
2. How did you help yourselves learn better? How did your teachers help?
3. What are the important factors to facilitate the development of your caring attributes through working in a small group?
4. According to your experience of participating in a small group, what were the difficulties or barriers influencing the development of your caring attributes and how did you manage them?

<u>Question Guide for educators</u>

Starting with the open-ended question:

Share how students can develop caring attributes through working in a small group for their nursing profession.

Probing questions:

1. With your experience of teaching students through a small group, what types of caring attributes students can be developed through this learning method?
2. How did you facilitate students to develop their caring attributes? Please share some strategies.
3. What are the important factors influencing the development of caring attributes among students through working in a small group?
4. According to your experience of teaching a small group, what were the difficulties or barriers influencing students' development of caring attributes and how did you help them manage those situations?
